# Treatment of metastatic ALK-positive non-small cell lung cancer: indirect comparison of different ALK inhibitors using reconstructed patient data

**DOI:** 10.3389/fonc.2025.1566816

**Published:** 2025-05-09

**Authors:** Vera Damuzzo, Lorenzo Gasperoni, Luna Del Bono, Andrea Ossato, Alessandro Inno, Andrea Messori

**Affiliations:** ^1^ Hospital Pharmacy, Vittorio Veneto Hospital, AULSS2 Marca Trevigiana, Vittorio Veneto, Italy; ^2^ Italian Society of Clinical Pharmacy and Therapeutics (SIFaCT), Turin, Italy; ^3^ Oncological Pharmacy Unit, IRCCS Istituto Romagnolo per lo Studio dei Tumori (IRST) “Dino Amadori”, Meldola, Italy; ^4^ Department of Pharmacy, School of Specialization in Hospital Pharmacy, University of Pisa, Pisa, Italy; ^5^ Scientific Committee, Italian Society of Clinical Pharmacy and Therapeutics, Turin, Italy; ^6^ Department of Pharmaceutical and Pharmacological Sciences, University of Padua, Padua, Italy; ^7^ Medical Oncology, Istituto di Ricovero e Cura a Carattere Scientifico (IRCCS) Ospedale Sacro Cuore Don Calabria, Negrar di Valpolicella, Italy; ^8^ Health Technology Assessment (HTA) Unit, Regional Health Service, Florence, Italy

**Keywords:** NSCLC, ALK-inhibitors, IPDfromKM, indirect comparison, Shiny method

## Abstract

**Introduction:**

Anaplastic lymphoma kinase (ALK) inhibitors (ALKi) are the standard treatment for metastatic, ALK-positive non-small cell lung cancer (NSCLC). Second- and third-generation ALKi, including alectinib, brigatinib, ensartinib, envonalkib, and lorlatinib, have shown better efficacy than crizotinib. However, due to the lack of direct head-to-head comparisons among these agents, the optimal treatment for metastatic ALK-positive NSCLC remains unclear.

**Methods:**

This study used the IPDfromKM (Individual Patient Data from Kaplan-Meier) method to reconstruct patient-level data from Kaplan-Meier curves of seven randomized phase III trials, involving a total of 3,850 patients. Crizotinib arms were pooled as the common comparator. Progression-free survival (PFS) was the primary endpoint, assessed using Cox proportional hazards models and restricted mean survival time (RMST). Subgroup analyses focused on patients with baseline central nervous system (CNS) metastases.

**Results:**

All ALKi significantly improved PFS compared to crizotinib. Lorlatinib showed the most meaningful improvement, with the greatest benefit in both overall PFS (HR=0.28; 95% CI 0.21-0.38) and CNS PFS (HR=0.09; 95% CI 0.04-0.2). In direct comparisons, lorlatinib outperformed brigatinib (HR=0.59; 95% CI 0.39-0.87) and envonalkib (HR=0.52; 95% CI 0.35-0.77) in terms of PFS. While lorlatinib also showed improved PFS compared to alectinib (HR=0.72; 95% CI 0.50–1.04) and ensartinib (HR=0.73; 95% CI 0.48–1.10), these differences were not statistically significant. Lorlatinib demonstrated the greatest benefit in PFS among patients with baseline CNS metastases.

**Conclusion:**

In this indirect comparison using reconstructed patient data, lorlatinib emerged as the most effective ALKi, showed the most favorable HR for PFS compared to the other ALKi, although it did not reach statistical significance versus alectinib and ensartinib. Additionally, lorlatinib showed the highest efficacy in the control of CNS progression.

## 1 Introduction

Non-Small Cell Lung Cancer (NSCLC) is a leading cause of cancer-related mortality worldwide (1).

Approximately 3–5% of NSCLC tumors harbor rearrangements of the anaplastic lymphoma kinase (ALK) gene, which defines a distinct molecular subtype primarily associated with adenocarcinoma histology. This subtype is more commonly observed in younger patients with no or limited smoking history and is characterized by a high tropism for the central nervous system (CNS) (2, 3).

Crizotinib, a first-generation ALK inhibitor (ALKi), has been the standard of care for metastatic ALK-positive NSCLC for several years. Its efficacy was demonstrated in the PROFILE 1014 phase III randomized trial, which showed a significant progression-free survival (PFS) benefit with crizotinib compared to platinum-based chemotherapy (10.9 vs. 7.0 months; HR 0.45, 95% CI 0.35–0.60) in patients with metastatic ALK-rearranged NSCLC (4, 5).

Since then, second-generation ALKi (alectinib, brigatinib, envonalkib, ensartinib) and the third-generation ALKi lorlatinib have been developed, demonstrating superior efficacy over crizotinib in terms of intracranial activity and PFS in randomized phase 3 clinical trials (6–14). However, in the absence of direct head-to-head trials comparing these agents, the relative efficacy of each drug remains unclear. The aim of this study is to perform a head-to-head treatment comparison analysis of different ALKi based on PFS results from randomized phase III trials the IPDfromKM method (15, 16). This approach enables the reconstruction of individual data points based on the Kaplan-Meier survival curves, facilitating cross-trial comparisons using reconstructed patient-level data. One of the key benefits of using IPDfromKM is its ability to indirectly assess time-to-event outcomes over extended follow-up periods, considering the exact time each event occurs. Furthermore, this method permits to pool data deriving from patients treated with the same regimen but enrolled in different RCTs. This pooling increases the sample size, accounts for variations in follow-up duration, and facilitates a more thorough evaluation of time-to-event outcomes. The survival curves for each regimen analyzed are then displayed on a multi-treatment Kaplan-Meier plot, offering a clear summary of the findings.

In addition, to investigate the ability to penetrate the central nervous system (CNS), a comparative sub-analysis of CNS progression outcomes is performed. Here we report the results of a comparative overview of different ALKi in patients with ALK-positive NSCLC who have received no prior treatment.

## 2 Materials and methods

### 2.1 Literature search

We searched the PubMed database to identify clinical trials for our analysis (last search on 01 October 2024). The search term was ((“Lung Neoplasms”[Mesh] OR “non-small cell lung cancer”[tiab] OR NSCLC[tiab] OR “lung cancer”[tiab]) AND (“Anaplastic Lymphoma Kinase”[Mesh] OR ALK[tiab] OR “ALK mutation”[tiab] OR “ALK-positive”[tiab]) AND (“first-line”[tiab] OR “first line”[tiab] OR “initial treatment”[tiab] OR “primary treatment”[tiab]) AND (“Antineoplastic Agents”[Mesh] OR “Protein Kinase Inhibitors”[Mesh] OR “targeted therapy”[tiab] OR “TKI”[tiab] OR crizotinib[tiab] OR alectinib[tiab] OR brigatinib[tiab] OR lorlatinib[tiab])). Our search identified 708 records. Clinical trials were selected using the automated flag filter options, resulting in 47 clinical trials. The main inclusion criteria were: (a) phase III trial; (b) first-line treatment of locally advanced or metastatic ALK-positive NSCLC; (c) TKI comparator arm (not chemotherapy); (d) PFS endpoint; (e) presented as a KM curve. For each included trial, we recorded the number of patients enrolled and the number of events (being either disease progression or death). To avoid duplicate inclusion of patients from the same trial, we considered the most recent publication.

### 2.2 Reconstructing patient-level data

The individual patient data (IPD) reconstruction method from Kaplan-Meier (KM) curves, known as IPDfromKM, was employed to derive individual patient data from the KM survival curves representing treatment and control arms across selected randomized clinical trials (RCTs) (15, 16).

Initially, the KM curves were digitized using WebPlotDigitizer (version 4.7, accessed online at https://apps.automeris.io/wpd/ on November 10, 2024). The digitized X and Y coordinates, along with the total number of patients and events, were inputted into the IPDfromKM software (version 1.2.3.0, last updated on March 22, 2022). The software then generated individual patient survival times (calculated as the time from enrolment to the last follow-up) and classified patient outcomes as alive, dead, or censored. This process yielded reconstructed patient-level data for each treatment arm of the RCT. In each trial, crizotinib was considered the treatment against which all other therapies were compared. Patients receiving crizotinib in the respective trials were pooled and constitute the common comparator of this analysis (control group).

### 2.3 Study design

The aim of this analysis was to determine which second or third-generation ALKi provides the best PFS when compared to crizotinib, which is the first-generation ALKi considered a standard of care during the design of RCT for novel ALKi. Kaplan-Meier PFS curves for each ALKi were compared both to each other and to a pooled control curve representing patients treated with crizotinib. Furthermore, we also analyze PFS in patients with brain metastasis at baseline. Restricted mean survival time (RMST) was calculated at two time points: 35 months on the overall population and 23 months when analyzing the cohort with brain metastasis at baseline.

### 2.4 Statistical analysis

To evaluate the efficacy of the various treatments, we used the Cox proportional hazards model to analyze PFS data, comparing each treatment to the pooled crizotinib control group. The outcomes were expressed as hazard ratios (HR) with 95% confidence intervals (95% CI). The homogeneity of the control groups was assessed through Likelihood ratio tests and concordance statistics. For indirect comparisons between the active treatments (covering all head-to-head comparisons), a Cox regression model was employed, along with the calculation of RMST. The statistical analyses were carried out using the survival package in R (version 4.3.2).

## 3 Results

**Figure 1:**
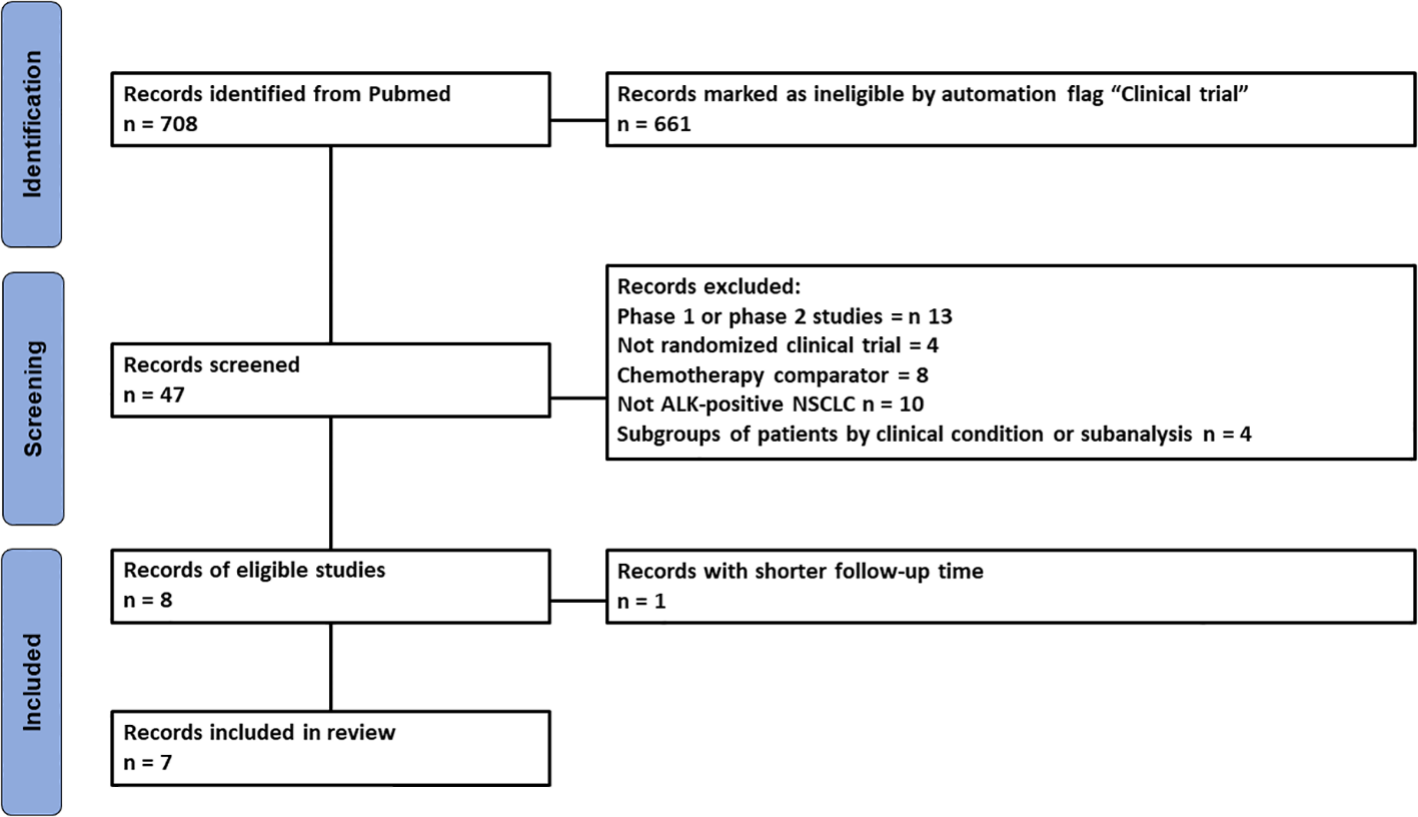
PRISMA flowchart of the process of trial selection. ALK, anaplastic lymphoma kinase; NSCLC, non-small cell lung cancer.

Literature search identified 708 records which were then selected according to our inclusion and exclusion criteria to identify the most recent randomized clinical trials (RCTs) investigating the efficacy of ALKi in patients with oncogene-driven NSCLC. The study selection process is shown in **Figure 1** according to PRISMA guidelines. Seven RCTs were identified for our analysis of indirect comparisons based on PFS endpoint.

Most patients had metastatic NSCLC and were treated with a second- or third-generation ALKi. Three trials (ALEX, J-ALEX and ALESIA) include patients treated with alectinib (6, 8, 9), while the remaining trials include the following inhibitors: ensartinib (eXalt-3) (10), lorlatinib (CROWN) (12), brigatinib (ALTA-1L) (13) and envonalkib (TQ-B3139-III-01) (14). All studies evaluated the efficacy of ALKi compared to crizotinib, which was used as the common comparator in our analysis. The results of literature search and the selection of are reported. The main clinical and demographic characteristics of the patients included in the trials are summarized in **Table 1**.

**Table 1:** The main clinical characteristics of patients treated with ALKi and included in the analysis are reported.

Trial	First Author, Year of Publication, Ref.	Treatments Arms	N. of Patients	% Asian patients	% Pt. CNS metastasis at baseline	% of Pt. prior chemotherapy*	Median Follow-Up	Median PFS	HR for PFS (95%CI)
**eXalt3**	Horn et al., 2021, (10)	Ensartinib vs crizotinib	247	61%	36%	25%*	23.8 20.2	not estimable 12.7	0.45 (0,30- 0.66)
**CROWN**	Solomon et al., 2023, (12)	Lorlatinib vs crizotinib	296	44%	39%	0%	36.7 29.3	not estimable 9.3	0.27 (0.18-0.39)
**ALTA-1L**	Camidge et al., 2021, (13)	Brigatinib vs crizotinib	275	39%	35%	27%	40.4 15.2	24.0 11.1	0.48 (0.35-0.66)
**ALEX**	Mok et al., 2020, (6)	Alectinib vs crizotinib	303	45%	40%	0%	37.8 23.0	34.8 10.9	0.43 (0.32-0.58)
**J-ALEX**	Hida et al., 2017, (8)	Alectinib vs crizotinib	207	100%	21%	36%	12.0 12.2	not estimable 10.2	0.34 (0.17-0.71)
**ALESIA**	Zhou et al., 2019, (9)	Alectinib vs crizotinib	187	100%	36%	0%	16.2 15.0	not estimable 10.7	0.37 (0.22-0.61)
**TQ-B3139-III-01**	Yang et al., 2023, (14)	Envonalkib vs crizotinib	264	100%	33%	25%	28.5 28.6	24.9 11.6	0.47 (0.34-0.64)

Pt, patients; CNS, central nervous system; Median follow-up time and median PFS are expressed in months, HR for PFS as reported in the original RCT. *Patients may have received up to one prior chemotherapy regimen for metastatic disease but were not required to have experienced disease progression during the treatment.

**Figure 2:**
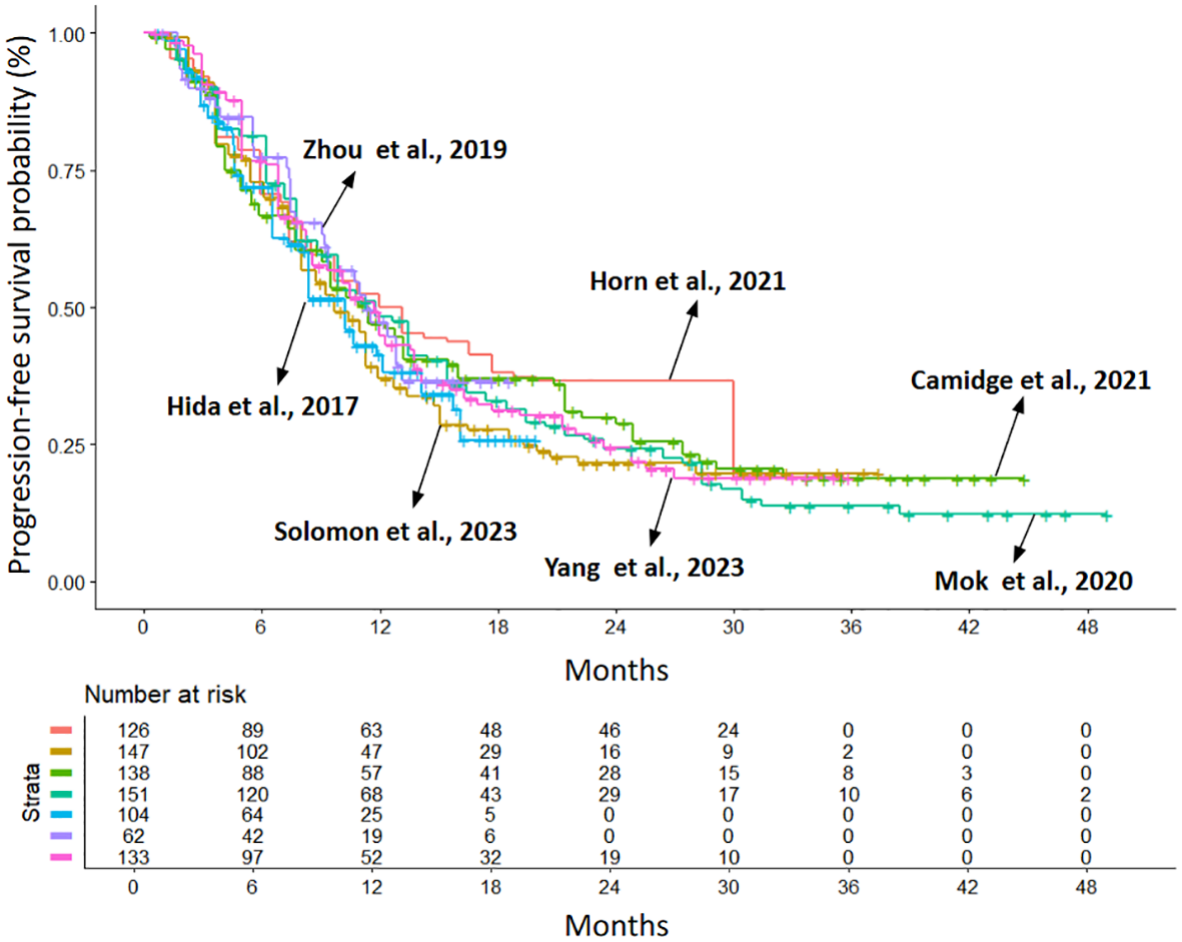
Kaplan–Meier curves generated after reconstructing patient-level data from the control arms of the included trials (crizotinib treated): eXalt-3 (n = 126; in red (10)); CROWN (n = 147; in gold (12)); ALTA-1L (n = 138; in light green (13)); ALEX (n = 151; in dark green (6)); J-ALEX (n = 104; in light blue (8)); ALESIA (n = 62; in purple (9)); TQ-B3139-III-01 (n = 133; in violet (14)). Endpoint: progression-free survival (PFS), time in months. n, number of patients.

The populations included in the studies are not homogeneous in terms of ethnicity, prior chemotherapy treatments, and the percentage of patients with CNS metastases. In fact, Asian patients enrolled across studies varied from 39% in ALTA-1 to 100% in J-ALEX, ALESIA and TQ-B3139-III-01 trials. Most patients received the treatment in the first-line setting for metastatic disease, but in 4 trials (J-ALEX, ALTA-1L, eXalt-3 and TQ-B3139-III-01) a proportion of patients (25 to 36%) had also received prior chemotherapy; finally, the incidence of baseline CNS metastases varies across studies, ranging from 21% to 40%. In view of these potential sources of heterogeneity in patient selection, we first performed a heterogeneity analysis to test whether patients enrolled in the different trials behaved similarly when treated with the same drug. To do this, we compared the PFS of the crizotinib arms of the different RCTs included in the analysis. We then performed a heterogeneity test. Despite the different clinical characteristics of the patients enrolled in the various studies, there were no significant differences between the HRs for PFS of the control arms and the heterogeneity of the results was low (likelihood ratio test=4.32 with 6 degrees of freedom, p=0.6). The Kaplan-Meier (KM) curves for PFS in the crizotinib arms overlap markedly, as shown in **Figure 2**.

**Figure 3:**
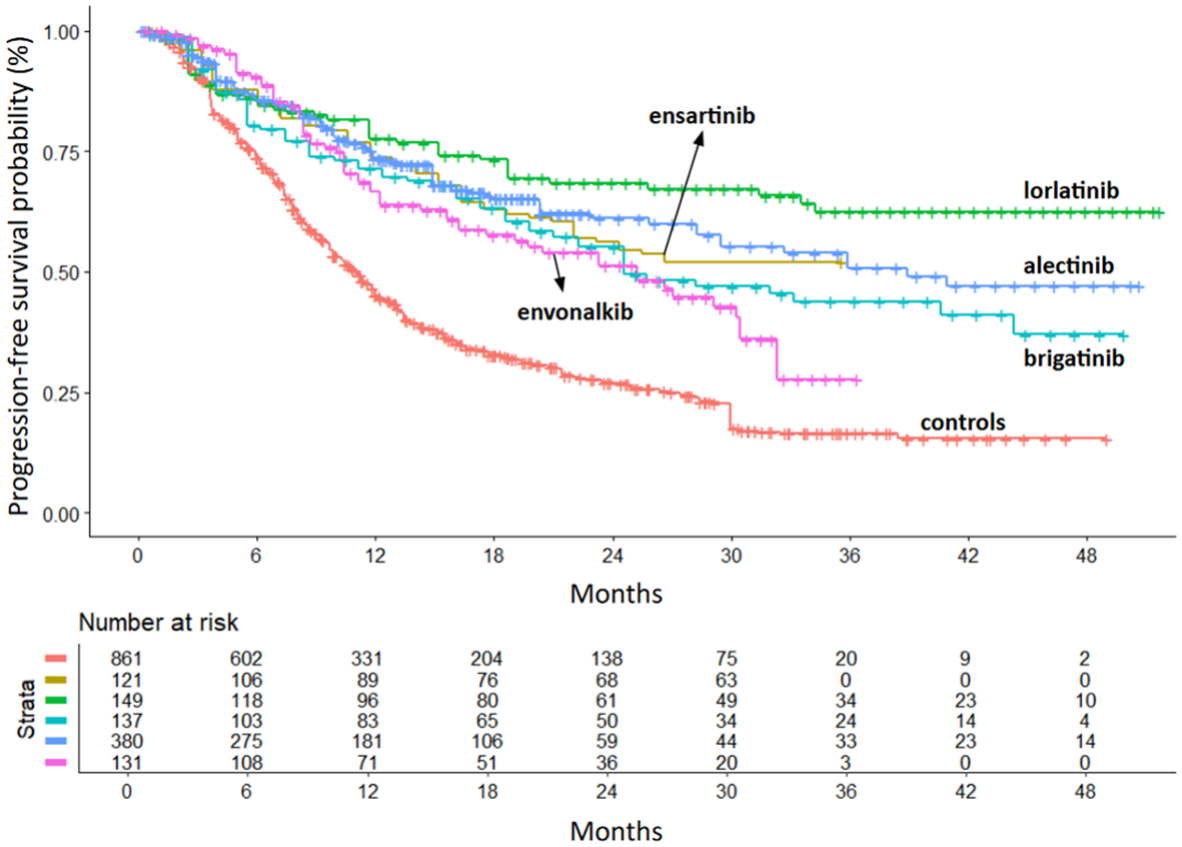
PFS of second- and third-generation ALKi compared to crizotinib controls. After reconstruction of individual patient data from seven trials, the following PFS KM curves were generated: crizotinib (n = 861; 7 cohorts (6, 8–10, 12–14); in red); ensartinib (n = 121 from eXalt-3 study (10); in gold); lorlatinib (n = 149 from CROWN study (12); in green); brigatinib (n = 137 from ALTA-1L study (13); in turquoise); alectinib (n = 380 from ALEX study (6), J-ALEX study (8) and ALESIA study (9); in blue); envonalkib (n = 131 from TQ-B3139-III-01 study (14); in pink). n, number of patients.

After ensuring the comparability of the RCTs, we compared the efficacy of second- and third-generation ALKi with crizotinib and with each other. The primary endpoint was PFS. All inhibitors included in the analysis showed a significant PFS advantage over crizotinib. PFS KM curves for each inhibitor are shown in **Figure 3**, with the KM curve from pooling crizotinib-treated patients shown in red.

**Figure 4:**
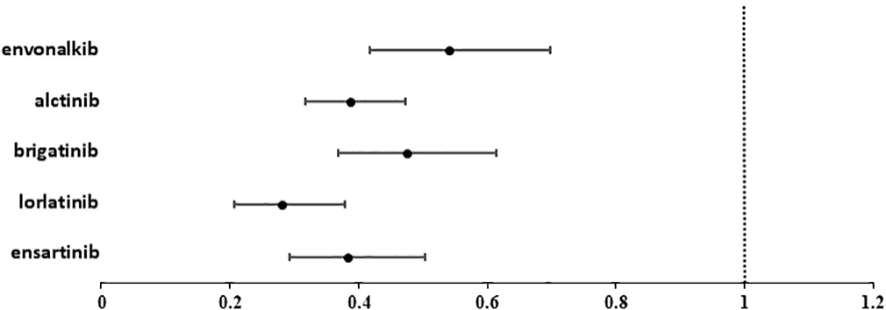
Forest plot showing the hazard ratio (HR) for PFS with 95% confidence interval (95%CI) of the different ALKi versus crizotinib.

Lorlatinib showed the greatest benefit (HR=0.28; 95%IC 0.21-0.38 vs. crizotinib). Ensartinib and alectinib showed similar efficacy, followed by brigatinib and envonalkib (**Table 2**, **Figure 4**).

**Table 2:** PFS estimates of second- and third-generation ALKi compared to crizotinib are reported as HR with 95% CI in the overall population and in patients with brain metastasis at baseline.

Overall population	HR	lower 95% CI	upper 95%CI
LORLATINIB	0.2797	0.2063	0.3792
ENSARTINIB	0.3832	0.292	0.503
ALECTINIB	0.3867	0.3167	0.4722
BRIGATINIB	0.4758	0.368	0.6151
ENVONALKIB	0.5397	0.4171	0.6984
Patients with brain metastasis at baseline	HR	lower 95% CI	upper 95%CI
LORLATINIB	0.09	0.04	0.2
ENVONALKIB	0.28	0.17	0.46
BRIGATINIB	0.34	0.23	0.51
ALECTINIB	0.36	0.25	0.52
ENSARTINIB	0.6	0.4	0.9

HR, hazard ratio; CI, confidence interval.

In the cross-treatment comparisons of HR for PFS, lorlatinib was significantly superior to brigatinib (HR= 0.59, 95%CI= 0.40-0.86) and envonalkib (HR= 0.52, 95%CI= 0.35-0.77) but not to alectinib (HR=0.72; 95%CI=0.50-1.04) and ensartinib (HR= 0.73, 95%CI= 0.49- 1.1). All other inter-treatment comparisons did not show significant differences, except for alectinib versus envonalkib, which showed superiority of alectinib (HR= 0.72, 95%CI= 0.52-0.99). Results are reported in **Table 3**.

**Table 3:** Inter-treatment comparisons of PFS for second- and third-generation ALKi are reported as HR with 95% CI in the overall population and in patients with brain metastasis at baseline.

Overall population	HR	lower 95% CI	upper 95%CI
LORLATINIB VS ENSARTINIB	0.73	0.485	1.098
LORLATINIB VS ALECTINIB	0.723	0.503	1.041
LORLATINIB VS BRIGATINIB	0.588	0.395	0.875
LORLATINIB VS ENVONALKIB	0.518	0.348	0.772
ENSARTINIB VS ALECTINIB	0.991	0.707	1.389
ENSARTINIB VS BRIGATINIB	0.805	0.554	1.171
ENSARTINIB VS ENVONALKIB	0.71	0.488	1.033
ALECTINIB VS BRIGATINIB	0.813	0.587	1.125
ALECTINIB VS ENVONALKIB	0.717	0.517	0.993
BRIGATINIB VS ENVONALKIB	0.882	0.613	1.269
Patients with brain metastasis at baseline	HR	lower 95% CI	upper 95%CI
LORLATINIB VS ENVONALKIB	0.321	0.125	0.828
LORLATINIB VS BRIGATINIB	0.265	0.108	0.65
LORLATINIB VS ALECTINIB	0.25	0.103	0.605
LORLATINIB VS ENSARTINIB	0.15	0.061	0.369
ENVONALKIB VS BRIGATINIB	0.824	0.435	1.558
ENVONALKIB VS ALECTINIB	0.778	0.419	1.443
ENVONALKIB VS ENSARTINIB	0.467	0.246	0.887
BRIGATINIB VS ALECTINIB	0.944	0.55	1.622
BRIGATINIB VS ENSARTINIB	0.567	0.321	1

HR, hazard ratio; CI, confidence interval.

Given that median PFS was not achieved for some of the inhibitors and that a significant proportion of patients-maintained disease control over time, we also performed a PFS analysis using restricted mean survival at 35 months (RMST). The results are shown in **Table 4**.

**Table 4:** PFS estimates of second- and third-generation ALKi compared to crizotinib are reported as RMST (expressed in months) with 95% CI in the overall population at 35 months of follow-up and at 48 months for RCT with an appropriate follow-up time.

Overall population: 35 months follow-up	RMST	lower 95% CI	upper 95%CI
LORLATINIB	26.536	24.395	28.677
ALECTINIB	24.756	23.229	26.283
ENSARTINIB	23.957	21.705	26.208
BRIGATINIB	22.821	20.544	25.098
ENVONALKIB	21.907	19.666	24.149
CRIZOTINIB	15.282	14.452	16.111
Overall population: 48 months follow-up	RMST	lower 95% CI	upper 95%CI
LORLATINIB	34.66	31.46	37.86
ALECTINIB	31.08	28.66	33.51
BRIGATINIB	28.16	24.8	31.51
CRIZOTINIB	16.83	15.41	18.24
Patients with brain metastasis at baseline: 23 months follow-up	RMST	lower 95% CI	upper 95%CI
LORLATINIB	20.5	18.44	22.55
ENVONALKIB	18.26	15.99	20.52
BRIGATINIB	17.74	15.42	19.66
ALECTINIB	15.71	13.66	17.75
ENSARTINIB	13.33	10.63	16.65
CRIZOTINIB	9.77	8.89	10.65

RMST, restricted mean survival time; CI, confidence interval.

PFS estimates of patients with brain metastasis at baseline are reported as RMST with 95% CI at 23 months of follow-up.

Once again, lorlatinib showed a significant advantage over crizotinib with an RMST of 26.5 months (95%CI 24.4-28.7) compared to 15.3 months of crizotinib (95%CI 14.5-16.1). Lorlatinib also showed a modest RMST advantage over alectinib (+1.78 months) and good advantages over ensartinib (+2.6 months), brigatinib (+3.7 months) and envonalkib (+4.6 months). The results are shown in **Table 4**. As long-term PFS results are available for brigatinib, alectinib and lorlatinib, we repeated the RMST analysis at 48 months. At this longer follow-up time, lorlatinib showed an RMST of 34.66 months (95%CI=31.46-37.86), twice as long as crizotinib, 3.6 months longer than alectinib and 6.5 months longer than brigatinib.

We then assessed whether second- and third-generation ALKi and crizotinib differed in controlling brain metastases. For patients with brain metastases at baseline, KM curves were available for all trials. However, there were subtle differences in outcome assessment. TheTQ-B3139-III-01 and CROWN trials consider only intracranial progression, the ALTA-1L trial combines intracranial progression with death from all causes, while the eXalt-3 and alectinib trials combine progression at all sites with death from all causes.

As these differences in outcome assessment may raise concerns about the comparability of the results, we first assessed whether patients with brain metastases enrolled in the different trials and treated with the same treatment (crizotinib) resulted in similar CNS PFS profiles. The median CNS PFS in the control arms ranged from a maximum of 10.6 months (95% CI 8.76-26.90) for patients in the J-ALEX trial to a minimum of 5.5 months (95% CI 4.16-7.68) for patients in the ALTA-1L trial. However, the inter-treatment comparison showed no significant differences in PFS between the two studies and the analysis of heterogeneity showed that although the definition of CNS PFS was slightly different across the studies, the results for patients treated with crizotinib were homogeneous (likelihood ratio test=8.96 with 5 degrees of freedom, p=0.1).

**Figure 5:**
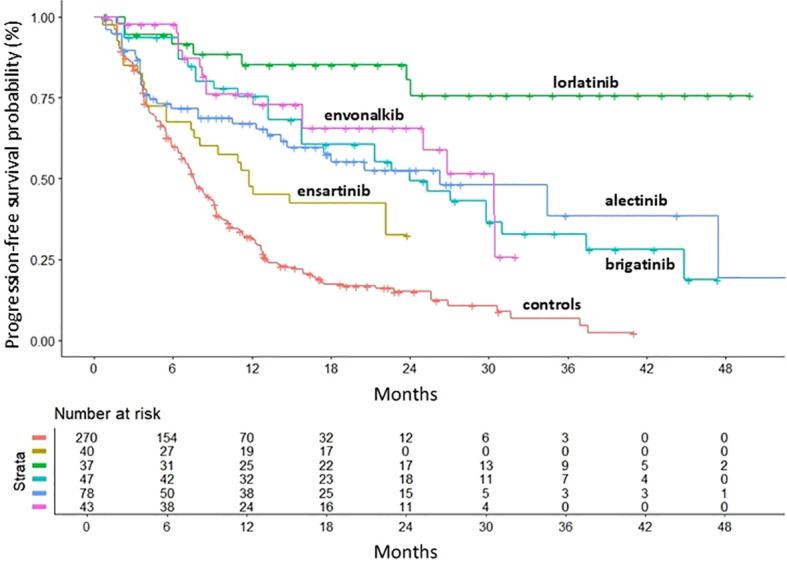
PFS of second- and third-generation ALKi compared to crizotinib controls in patients with brain metastasis at baseline. PFS KM curves are reported: crizotinib (n = 270; 7 cohorts (6, 8–10, 12–14); in red); ensartinib (n = 40 from eXalt-3 study (10); in gold); lorlatinib (n = 37 from CROWN study (12); in green); brigatinib (n = 47 from ALTA-1L study (13); in turquoise); alectinib (n = 78 from J-ALEX study (8); in blue); envonalkib (n = 43 from TQ-B3139-III-01 study (14); in pink). n, number of patients.

When we looked at the efficacy of the different active treatments in the selected RCTs, lorlatinib was significantly superior to crizotinib (HR=0.09, 95%CI 0.05-0.2) and all other ALKi in the control of brain metastases (**Figure 5**). The HR for PFS in patients with brain metastases at baseline compared to crizotinib is shown in **Table 2**, while the results of the comparison between treatments are shown in **Table 3**.

Envonalkib ranked second (HR vs. crizotinib = 0.28, 95%CI 0.11-0.46). However, its efficacy in patients with brain metastases was significantly higher only when compared to ensartinib (HR= 0.47, 95%CI= 0.25-0.89). Brigatinib and alectinib showed a similar benefit compared to crizotinib, with a reduction of 65% of the risk of progression, but no significant differences between them. This trend was also confirmed when CNS PFS was assessed by RMST at 23 months. Lorlatinib was the most effective treatment for controlling brain metastases in ALK-mutated NSCLC patients with an RMST of 20.5 months (95%CI=18.44-22.55 months), followed by envonalkib and brigatinib which showed similar RMST with 2 months less than lorlatinib. Alectinib and ensartinib showed shorter RMST, with a benefit of 4.8 and 7.7 months, respectively, compared to crizotinib.

## 4 Discussion

The standard first-line treatment for patients with metastatic ALK-positive NSCLC consists of an ALKi. Several second- and third-generation ALKi, including alectinib, brigatinib, ensartinib, envonalkib, and lorlatinib, have demonstrated superiority over the first-generation inhibitor crizotinib. However, due to the lack of head-to-head comparative studies among these agents, definitive conclusions cannot be drawn regarding the optimal first-line treatment.

In the present analysis, based on reconstructed patient data from randomized phase III trials, lorlatinib emerged as the ALKi with the most favorable HR for PFS compared to all other ALKi, with a RMST of 34.66 months at a follow-up of 48 months, although the difference in PFS between lorlatinib and alectinib or ensartinib was not statistically significant. A critical aspect of ALK-positive NSCLC is its tropism for the CNS. Unfortunately, the development of brain metastases is associated with a poor prognosis and, if symptomatic, can significantly impair quality of life. For this reason, the prevention and management of brain metastases are crucial in the treatment of ALK-positive NSCLC. In this context, lorlatinib demonstrated the highest efficacy in controlling CNS disease in the present analysis.

However, differences in efficacy must always be evaluated in the context of the specific toxicity profiles of the various treatments. In this regard, a key limitation of our analysis is its exclusive focus on efficacy endpoints, without consideration of toxicity. However, some speculations can be made based on the safety data reported in RCT. In fact, regarding the safety profiles of the three ALKi with the most favorable PFS HRs compared to crizotinib, RCT reported the following incidences of treatment-related grade ≥3 adverse events: 67% among patients treated with lorlatinib, 52% among those treated with alectinib (in the international ALEX study), and 5.04% among those treated with ensartinib. Treatment discontinuation rates due to adverse events were 7% for lorlatinib, 11% for alectinib and 9.1% for ensartinib (6, 8–10). The toxicity profiles differ significantly among these agents: lorlatinib was associated with hypercholesterolemia (any grade, 70%; G3-G4, 16%), hypertriglyceridemia (any grade, 64%; G3-G4,20%), edema (any grade, 55%; G3-G4, 4%), increased weight (any grade, 38%; G3-G4, 17%), cognitive effects (any grade, 21%; G3-G4, 2%) and hypertension (any grade, 18%; G3-G4, 10%) (11); alectinib was associated with anemia (any grade, 20%; G3-G5, 7%), edema (any grade, 17%; G3-G5 0%), ALT increased (any grade, 15%; G3-G4 5%), AST increased (any grade, 14%; G3-G5, 5%), and bilirubin increased (any grade, 15%; G3-G5, 2%), as reported in the international ALEX trial (6); ensartinib was associated with rash (any grade, 67.8%; G3-G4, 11.2%), ALT increased (any grade, 48.3%; G3-G4, 4.2%), AST increased (any grade, 37.8%; G3-G4, 0.7%), pruritus (any grade, 26.6%; G3-G4, 2.1%), constipation (any grade, 20.3%; grade 3-4, 0%), mostly G1-G2 (10).

Another critical aspect not addressed in our analysis is the impact of subsequent lines of therapy on overall survival. For instance, patients who progress on second-generation ALKi such as alectinib may still derive significant benefit from lorlatinib. A phase II trial reported a response rate of 29%, an intracranial response rate of 53%, and a median PFS of 6.9 months with lorlatinib in patients previously treated with two or three ALKi (17). Conversely, for patients progressing on lorlatinib, current treatment options are limited to platinum-based doublet chemotherapy (18), although novel ALKi are under development to address resistance mutations associated with lorlatinib (19). To assess the impact of subsequent lines of treatment on patient outcomes, OS would have been the preferred endpoint. However, OS data from RCTs for some ALK, including lorlatinib, envonalkib and ensartinib, are still immature, and Kaplan-Meier OS curves are not yet available for an IPDfromKM-based analysis (10, 12, 14). Therefore, a meaningful OS analysis will require more mature data. In the absence of reliable OS comparisons, it is unclear whether the optimal strategy for advanced ALK-positive NSCLC is upfront use of a third-generation ALK TKI or a sequential approach with a second-generation inhibitor followed by lorlatinib at progression. However, data from first-line RCTs show that at least 40% of patients do not receive further treatment after progression, limiting the feasibility of a sequencing approach to about 60% of cases. This highlights the importance of selecting the first-line treatment with the best PFS (20).

Further efforts in research should be made to personalize first-line treatment based on potential predictive biomarkers of efficacy and primary resistance to ALK inhibitors. Several studies suggest that the ALK fusion variant and co-mutations may influence clinical outcomes. In particular, TP53 mutations and the EML4-ALK v3 variant have been associated with a poorer prognosis (21). Among second- and third-generation inhibitors, in the absence of direct comparisons and recognizing the limitations of indirect comparisons across studies, the longest reported PFS for patients with the EML4-ALK v3 variant is 60 months with lorlatinib, compared to 16–18 months with alectinib and brigatinib (13, 22, 23). In patients with TP53 mutations, PFS was 51.6 months with lorlatinib, whereas it was only 18 months with brigatinib (13, 23). These findings suggest that in patients with molecular features associated with greater aggressiveness, such as the EML4-ALK v3 variant or TP53 mutations, first-line treatment with lorlatinib may be preferable to second-generation inhibitors.

The IPDfromKM method used in this analysis offers significant advantages by reconstructing individual patient data from Kaplan-Meier (KM) curves, and is increasingly being used in the field of survival indirect comparisons in oncology (24–27). First, this method allows crizotinib-treated patients to be pooled, thus increasing the sample size of the common comparator, while maintaining randomization as each patient in the active arms found their randomized counterpart in the control arm. Secondly, a key advantage is that it preserves the exact timing of events, which is often lost in binary meta-analyses that rely on simplified measures such as odds ratios. In addition, the multi-KM plot resulting from the comparison between treatments provides a clear and effective way to communicate the results, making them easy to interpret for both researchers and clinicians. One of the main limitations of survival analysis based on the IPDfromKM method is its dependence on the availability of subgroup-specific KM curves. Without these subgroup curves in the original studies, it becomes difficult to perform survival analyses for specific patient subgroups. However, in our context, it was also possible to analyze the efficacy of ALKi in a cohort of patients with CNS metastases at baseline, as KM curves were available. Some approximations were made as disease progression was expressed in slightly different ways in the selected RCTs, but the positive results of the heterogeneity analysis allowed for overall comparability of the results.

In conclusion, in our indirect comparison of second- and third-generation ALKi, lorlatinib achieved the best HR for overall PFS compared to the other ALKi, although it did not reach statistical significance versus alectinib and ensartinib, and the best HR for CNS PFS. However, in daily clinical practice, clinicians should carefully evaluate the balance between efficacy and side-effect profiles and determine the most appropriate treatment sequence, taking into account the characteristics and preferences of the individual patient. Despite some limitations, the IPDfromKM method is a powerful and easy-to-use tool for performing indirect treatment comparisons, which improves the understandability of indirect comparative survival analyses when individual patient data are not available.

## Data Availability

The raw data supporting the conclusions of this article will be made available by the authors, without undue reservation.
